# Requirement for hippocampal CA3 NMDA receptors in artificial association of memory events stored in CA3 cell ensembles

**DOI:** 10.1186/s13041-023-01004-2

**Published:** 2023-01-20

**Authors:** Masanori Nomoto, Noriaki Ohkawa, Kaoru Inokuchi, Naoya Oishi

**Affiliations:** 1 Research Centre for Idling Brain Science, University of Toyama, Toyama, 930-0194 Japan; 2 Department of Biochemistry, Graduate School of Medicine and Pharmaceutical Sciences, University of Toyama, Toyama, 930-0194 Japan; 3 CREST, JST, University of Toyama, Toyama, 930-0194 Japan; 4 Division for Memory and Cognitive Function, Research Center for Advanced Medical Science, Comprehensive Research Facilities for Advanced Medical Science, Dokkyo Medical University, Shimotsuga-Gun, Tochigi, 321-0293 Japan; 5 Present Address: Pharmaceutical Division, Pharmaceutical Research Laboratory, Drug Discovery and Pharmacology Group, Ube Corporation, 1978-5, Kogushi, Ube, Yamaguchi 755-8633 Japan

**Keywords:** Hippocampus, CA3, NMDA receptor, Recurrent circuit, Artificial association, Synaptic plasticity, Optogenetics, Engram, Synthetic memory, False memory

## Abstract

The *N*-methyl-d-aspartate receptors (NRs) in hippocampal CA3 are crucial for the synaptic transmission and plasticity within the CA3 recurrent circuit, which supports the hippocampal functions, such as pattern completion, and reverberatory association of sensory inputs. Previous study showed that synchronous activation of distinct cell populations in CA3, which correspond to distinct events, associated independent events, suggesting that the recurrent circuit expressing NRs in CA3 mediates the artificial association of memory events stored in CA3 ensembles. However, it is still unclear whether CA3 NRs are crucial for the artificial association of memory events stored in the CA3 ensembles. Here we report that the triple transgenic mice (cfos-tTA/KA1-Cre/NR1 flox/flox), which specifically lack NRs in the CA3 cell ensembles, showed impairment in artificial association between two events, which in control mice triggered artificial association. This result indicates that NRs in the hippocampal CA3 are required for the artificial association of memory events stored in the CA3 cell ensembles.

**Supplementary Information:**

The online version contains supplementary material available at 10.1186/s13041-023-01004-2.

## Introduction

The hippocampus plays a central role in learning and memory to process and associate multimodal sensory and episodic information [1]. Previous studies showed that the *N*-methyl-d-aspartate receptors (NRs) that express broadly in the central nervous system are crucial for regulation of excitatory synaptic transmission, synaptic plasticity, and some forms of cognitive functions [2]. NRs in dentate gyrus (DG) are involved in pattern separation [3] and NRs in CA3 are involved in the reverberatory association of sensory inputs [4] and pattern completion [5]. NRs in CA1 are involved in the memory acquisition [6], indicating that NRs in the hippocampus are essential for multiple brain functions. Within the DG-CA3-CA1 trisynaptic circuit of hippocampus, the CA3 forms extensive interconnections within CA3, that is called as a recurrent circuit [7] where NRs are required for excitatory transmission and long-term potentiation (LTP) among CA3-CA3 synapses. Theoretical models have suggested that the CA3 recurrent circuit implemented with NR function is capable of preserving multiple information to generate a new associative memory [8, 9]. This implies that the simultaneous and artificial reactivation of specific cell ensembles in CA3 corresponding to pre-stored distinct memories enables to induce the artificial association that is a new link between distinct memories and an acquisition of a new memory. To test this possibility, we have established the optogenetic technique to specifically manipulate CA3 ensemble corresponding to the behavioral event [10, 11]. We showed that activation of CA3 ensemble recalls the memory [11] and that synchronous activation of distinct cell populations in CA3, which correspond to distinct events links these initially independent events [10]. These findings suggest that the CA3 recurrent circuit expressing NRs mediates artificial association of memory events stored in CA3 ensembles. However, it is still unclear whether CA3 NRs are crucial for the artificial association of memory events stored in the CA3 ensembles. In this study, we aim to clarify the role of CA3 NRs in the artificial association, which would provide an important insight into the hippocampal CA3 recurrent circuit in learning and memory.

## Results

**Fig. 1:**
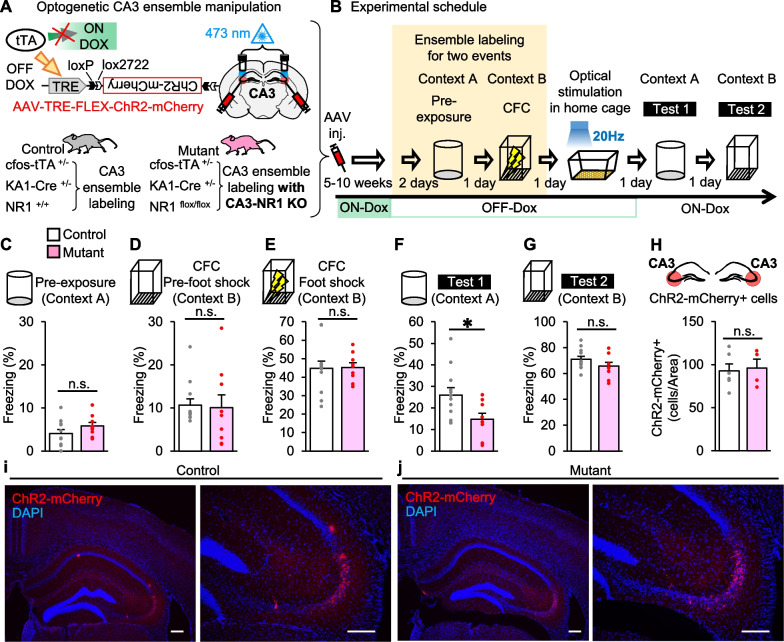
Deficit of CA3 NRs impaired in artificial association of memory events by CA3 ensemble activation. **a** CA3-specific cell ensemble labeling for CA3-NR1 KO mutant and control animals. Mice were injected with the AAV vector and implanted with guide cannulas targeting bilateral CA3 regions. In the absence of doxycycline (Dox), tetracycline transactivator (tTA) binds to the tetracycline-responsive element (TRE) to drive the expression of the target gene specifically in the CA3 region of the hippocampus. Therefore, the subpopulation of cells that expressed Cre recombinase in CA3 and activated during learning will express ChR2-mCherry. AAV injection coordinate and cannula placement targeting CA3 are shown. Gray and white triangles represent loxP and lox2272 sequences, respectively. **b** Experimental schedule. After bilateral CA3 infection with the AAV, the control or mutant mice were exposed to two events in OFF Dox condition. One day after CFC, the CA3 regions of the control group (*n* = 12) and mutant group (*n* = 9) were optically stimulated. Mice were tested for their freezing responses in contexts A and B on 1 and 2 days after the optical stimulation, respectively. **c–g** Columns showing freezing responses during (**c**) pre-exposure session in context A (two-sided unpaired Student’s *t* test: *t*_19_ = 1.359, *p* = 0.1901), **d** pre-foot shock session during CFC in context B (unpaired Student’s *t* test: *t*_19_ = 0.1939, *p* = 0.8483), **e** CFC foot shock session in context B (unpaired Student’s *t* test: *t*_19_ = 0.09743, *p* = 0.9234), **f** test session in context A (unpaired Student’s *t* test: *t*_19_ = 2.453, *p* = 0.024), **g** test session in context B (unpaired Student’s *t* test: *t*_19_ = 1.441, *p* = 0.1659). **h** Averaged numbers of ChR2-mCherry-positive cells per section in the CA3 region from control (*n* = 6) and mutant mice (*n* = 4) (counted from both left and right CA3 area, two sections/animal, unpaired Student’s *t* test: *t*_8_ = 0.2532, *p* = 0.8065). **i**, **j** Representative ChR2-mCherry expression images in CA3 of (**i**) control and (**j**) mutant animal. Left, ×4 magnification images. Right, ×10 magnification images. Scale bar, 200 μm. Mice were exposed to the two events for ensembles labeling in the OFF Dox condition. *p < 0.05; n.s., no significant difference between the two groups; error bars are the means ± SEMs

We generated triple transgenic mice (cfos-tTA/KA1-Cre/NR1 flox/flox) as test group (mutant) that enable to label CA3 ensemble and specifically lack NRs in CA3 by crossing the transgenic mice cfos-tTA/KA1-Cre [10, 11] and CA3-NR1 KO [4, 5] and set the double transgenic mice (cfos-tTA/KA1-Cre/NR1+/+) that normally express NRs as control group (control) (Fig. 1a). We used the AAV vector expressing a light-activated cation channel, channelrhodopsin-2 (ChR2) under the control of the *c-fos* gene promoter, in Cre recombinase expression-dependent manner (see Additional file 1 for detailed method).

After AAV injection and cannula implantation [10–12] (Fig. 1b), the mice were taken off Dox for 2 days, then subjected to context A pre-exposure and contextual fear conditioning (CFC) in context B (referred to as “two events”) to manipulate CA3 ensembles corresponding to these two events as reported previously [10, 11] (Fig. 1b). During these sessions, control and mutant mice showed comparable freezing (Fig. 1c–e). One day after CFC, mice were subjected to 20-Hz optical stimulation session to synchronously activate labelled CA3 cell ensembles [10, 13]. Our previous studies showed that the artificial activation of CA3 ensembles induced the recall [10] and association of specific memories [11].

Consistent with previous result [10], after the artificial activation, control and mutant mice showed significantly high freezing in context A, where they had not experienced CFC compared to the freezing in context A pre-exposure session (two-sided paired Student’s *t* test for context A pre-exposure vs. test in context A: control, *t*_11_ = 6.878, *p* = 2.6E−05; mutant, *t*_8_ = 3.199, *p* = 0.012) (Fig. 1f). Mutant mice showed significantly less freezing than control mice in context A (Fig. 1f) but not context B (Fig. 1g). The number of ChR2-mCherry-positive cells was comparable between genotypes (Fig. 1h–j), suggesting that the incomplete artificial memory in mutant mice was not due to decrease in labeling efficiency and that the deficiency of CA3 NRs does not affect the efficacy of CA3 ensemble labeling. These results indicate that artificial association of memory events encoded by distinct ensembles in CA3 depends on the CA3 NRs.

## Discussion

We detected the NRs deficiency in CA3 failed the artificial association of memory events by optogenetic activation of CA3 ensembles. Our result indicates that the NRs-dependent excitatory transmission and/or synaptic plasticity in the CA3 recurrent circuit is crucial for the artificial association of memories stored in CA3. Our finding supports the hypothesis that the recurrent circuit plays a role as an associator of memories stored separately by preserving and associating the multiple information within the recurrent circuit [8, 9]. New connection between two distinct CA3 ensembles rather than connection between CA3 ensemble and non-ensemble CA3 cells may be important for the artificial association between distinct memories because these labelled ensembles might be directly activated and preferentially wired compared to other CA3 cells during the optogenetic activation.

Subpopulation of CA3 neurons that are activated during CFC learning specifically reactivates during re-exposure to the learnt context and during memory recall [14]. Thus, our labeling protocol for two events may label the distinct ensembles corresponding to two events. The deficit of NRs in CA3 leads to impairments of 20-Hz optical in vivo LTP within the CA3 area [10], and reverberatory activity in CA3 and CA1 areas that contribute to integration of sensory inputs [4]. Thus, not only CA3 NRs-dependent LTP within the recurrent circuit but also CA3 NRs-dependent reverberatory activity may underly the artificial association of memories stored in CA3. Under the physiological condition, the NRs in CA3 are involved in the association of sensory inputs [4] and might be also involved in the acquisition of the temporal order memory, which is a temporal association of successive events and dependent on the activity of CA3 region [15].

Further studies are required to clarify whether other brain areas forming a recurrent circuit, such as cortical area, also have the same ability to CA3 and whether the recurrent circuit has the unique function to associate multimodal information and episodes compared to the other brain areas.

## Supplementary Information

**Additional file 1.** Detailed methods.

## Data Availability

The datasets used and/or analyzed during the current study are available from the corresponding author on reasonable request.

## Abbreviations

NMDA: *N*-Methyl-d-aspartate
KO: Knock-out
KA1: Kainite receptor subunit 1
NR1: NMDA receptor subunit 1
DG: Dentate gyrus
LTP: Long-term potentiation
flox: Flanked by LoxP sequence
ChR2: Channelrhodopsin-2
Dox: Doxycycline
AAV: Adeno-associated virus
tTA: Tetracycline transactivator
TRE: Tetracycline-responsive element
CFC: Contextual fear conditioning
