# Correction: Requirement for hippocampal CA3 NMDA receptors in artificial association of memory events stored in CA3 cell ensembles

**DOI:** 10.1186/s13041-023-01016-y

**Published:** 2023-03-13

**Authors:** Masanori Nomoto, Noriaki Ohkawa, Kaoru Inokuchi, Naoya Oishi

**Affiliations:** 1 Research Centre for Idling Brain Science, University of Toyama, Toyama, 930‑0194 Japan; 2 Department of Biochemistry, Graduate School of Medicine and Pharmaceutical Sciences, University of Toyama, Toyama, 930‑0194 Japan; 3 CREST, JST, University of Toyama, Toyama, 930‑0194 Japan; 4 Division for Memory and Cognitive Function, Research Center for Advanced Medical Science, Comprehensive Research Facilities for Advanced Medical Science, Dokkyo Medical University, Shimotsuga‑Gun, Tochigi 321‑0293 Japan; 5 Present Address: Pharmaceutical Division, Pharmaceutical Research Laboratory, Drug Discovery and Pharmacology Group, Ube Corporation, 1978‑5, Kogushi, Ube, Yamaguchi 755‑8633 Japan

**Correction** **:** **Molecular Brain (2023) 16:12.** https://doi.org/10.1186/s13041-023-01004-2

Following publication of the original article [1], the authors identified a typesetting mistake in Fig. 1 (panel B). The correct Fig. 1 is included in this Correction and the original article has been corrected.

**Fig. 1:**
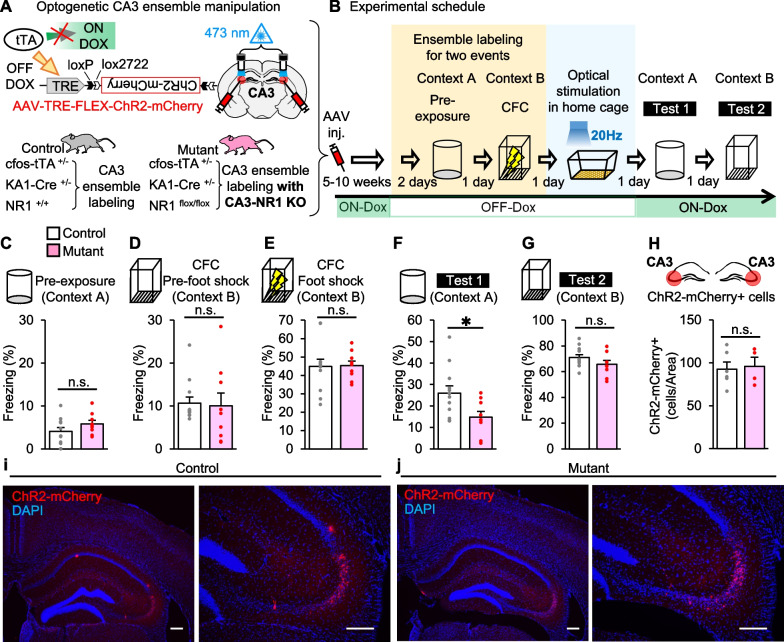
Deficit of CA3 NRs impaired in artificial association of memory events by CA3 ensemble activation. **a** CA3-specific cell ensemble labeling for CA3-NR1 KO mutant and control animals. Mice were injected with the AAV vector and implanted with guide cannulas targeting bilateral CA3 regions. In the absence of doxycycline (Dox), tetracycline transactivator (tTA) binds to the tetracycline-responsive element (TRE) to drive the expression of the target gene specifically in the CA3 region of the hippocampus. Therefore, the subpopulation of cells that expressed Cre recombinase in CA3 and activated during learning will express ChR2-mCherry. AAV injection coordinate and cannula placement targeting CA3 are shown. Gray and white triangles represent loxP and lox2272 sequences, respectively. **b** Experimental schedule. After bilateral CA3 infection with the AAV, the control or mutant mice were exposed to two events in OFF Dox condition. One day after CFC, the CA3 regions of the control group (*n* = 12) and mutant group (*n* = 9) were optically stimulated. Mice were tested for their freezing responses in contexts A and B on 1 and 2 days after the optical stimulation, respectively. **c**–**g** Columns showing freezing responses during **c** pre-exposure session in context A (two-sided unpaired Student’s *t* test: *t*_19_ = 1.359, *p* = 0.1901), **d** pre-foot shock session during CFC in context B (unpaired Student’s *t* test: *t*_19_ = 0.1939, *p* = 0.8483), **e** CFC foot shock session in context B (unpaired Student’s *t* test: *t*_19_ = 0.09743, *p* = 0.9234), **f** test session in context A (unpaired Student’s *t* test: *t*_19_ = 2.453, *p* = 0.024), **g** test session in context B (unpaired Student’s *t* test: *t*_19_ = 1.441, *p* = 0.1659). **h** Averaged numbers of ChR2-mCherry-positive cells per section in the CA3 region from control (*n* = 6) and mutant mice (*n* = 4) (counted from both left and right CA3 area, two sections/animal, unpaired Student’s *t* test: *t*_8_ = 0.2532, *p* = 0.8065). **i**, **j** Representative ChR2-mCherry expression images in CA3 of (**i**) control and (**j**) mutant animal. Left, ×4 magnification images. Right, ×10 magnification images. scale bar, 200 μm. Mice were exposed to the two events for ensembles labeling in the OFF Dox condition. *p < 0.05; n.s., no significant difference between the two groups; error bars are the means ± SEMs

The publisher apologises to the authors and readers for the inconvenience caused by the error.
