# Supplementary material for: Requirement for hippocampal CA3 NMDA receptors in artificial association of memory events stored in CA3 cell ensembles
Source: Mol Brain. 2023 Jan 20;16:12. doi: 10.1186/s13041-023-01004-2 (PMC9854113; doi:10.1186/s13041-023-01004-2)
Supplement: Supplementary file 1 — Additional file 1. Detailed methods. [file 13041_2023_1004_MOESM1_ESM.docx]

**Additional file 1**

**Requirement for hippocampal CA3 NMDA receptors in artificial association of memory events stored in CA3 cell ensembles**

**Masanori Nomoto^1,2,3†✱^, Noriaki Ohkawa^4^, Kaoru Inokuchi^1,2,3✱^, Naoya Oishi^1,2,3†^**

†These authors contributed equally: Masanori Nomoto, Naoya Oishi.

*Correspondence authors: M.N. (email: nomoto@med.u-toyama.ac.jp) or K.I. (email: inokuchi@med.u-toyama.ac.jp)

**Email addresses**

Masanori Nomoto: nomoto@med.u-toyama.ac.jp

Noriaki Ohkawa: nohkawa@dokkyomed.ac.jp

Kaoru Inokuchi: inokuchi@med.u-toyama.ac.jp

Naoya Oishi: oishi708@gmail.com

**METHODS**

**Animals**

The c-fos-tetracycline transactivator (cfos-tTA) mice were purchased from the Mutant Mouse Regional Resource Center (stock no. 031756-MU). The KA1-Cre mice were purchased from Jackson Laboratory (Jackson Laboratory stock no: 006474, G32-4 Cre). Floxed-NR1 mice (Jackson Laboratory stock no: 005246) were donated by Drs. S. Tonegawa (RIKEN-Massachusetts Institute of Technology) and S. Itohara (RIKEN Brain Science Institute).

The cfos-tTA/KA1-Cre double transgenic mice were generated via *in vitro* fertilization with eggs from C57BL/6J mice and embryo transfer techniques as described previously [10, 11]. The CA3 pyramidal cell-restricted *N*-methyl-D-aspartate (NMDA) receptor knock-out/c-fos-tTA (cfos-tTA/KA1-Cre/NR1 flox/flox) mice were generated from c-fos-tTA/KA1-Cre double transgenic mice and homozygous floxed-NR1 mice via *in vitro* fertilization [4]. All experimental mice were C57BL/6J background as described previously [10, 11]. Male mice were used for all the experiment.

The mice were maintained on a 12-h light-dark cycle (lights on 8:00 am) at 24 ± 3°C and 55 ± 5% humidity with food and water *ad libitum* and were housed with littermates until the surgeries. All procedures involving the use of animals complied with the guidelines of the National Institutes of Health and were approved by the Animal Care and Use Committee of the University of Toyama.

**Viral vectors**

pAAV-TRE2G::DIO-ChR2(T159C)-mCherry plasmid and this recombinant AAV vectors were produced as described previously [10, 11]. Mice were injected with AAV9-TRE2G::DIO-ChR2(T159C)-mCherry at a titer of 1.3 × 10^13^ viral genomes (vg)/ml.

**Stereotactic surgery and cannula placement**

Surgeries were carried out as described previously [10, 11]. The mice were anesthetized with isoflurane, given intraperitoneal injections of medetomidine hydrochloride (0.75 mg/kg of body weight), midazolam (4 mg/kg of body weight), and butorphanol tartrate (5 mg/kg of body weight) and then placed in a stereotactic apparatus (Narishige, Tokyo, Japan). Then, 0.3 μl of AAV9-TRE2G::DIO-ChR2(T159C)-mCherry was bilaterally injected into the CA3 region (anterior-posterior [AP], −2.0 mm; medial-lateral [ML], ±2.3 mm from bregma; doral-ventral [DV], −2.0 mm from dura) using a glass micropipette filled with mineral oil attached to a 10-μl Hamilton microsyringe. A microsyringe pump (Narishige, Tokyo, Japan) and its controller were used to control the speed of the injection (0.1 μl/min). The needle was slowly lowered to the target site and remained there for 3 min after the injection. Then, stainless steel guide cannulas (internal diameter, 0.29 mm; outer diameter, 0.46 mm; Plastics One, Roanoke, VA) were bilaterally implanted in the CA3 areas (AP, −2.0 mm; ML, ±2.3 mm; DV, −1.0 mm from bregma). Microscrews were anchored in the skull near bregma and lambda, and the guide cannulas were fixed in place with dental cement. After the surgery, dummy cannulas with caps were inserted into the guide cannulas as protective covers. Mice were 12–20 weeks old at the time of surgery, and were allowed to recover 5–10 weeks before being used in behavioral experiments.

**Behavioral analyses and optical stimulation**

Behavioral analysis was conducted as described previously [10]. The home cage of each mouse was placed on a desk in the animal housing room for approximately 10 min before being transferred to the adjoining experimental room. For behavioral tests, each mouse was gently caught at the base of its tail and transferred to each context described below.

Context A was a cylindrical chamber (diameter, 180 mm; height, 230 mm) with a white acrylic floor and walls covered with black tape. Context B was a square-type chamber (175 × 165 mm; height, 300 mm) with a transparent acrylic board front, white sides and back walls, and a floor consisting of 26 stainless steel rods with a diameter of 2 mm placed 5 mm apart with a scented tray containing 0.25% benzaldehyde underneath. The rods were connected to a shock generator via a cable harness. Constant minimal illumination was provided by a small light in the chamber. The room lights were off for context B but on for contexts A.

The mice were 12–20 weeks old at the time of surgery, and were allowed to recover 5–10 weeks before being used in behavioral experiments. Cannula-implanted and AAV-injected mice were maintained on 40 mg/kg Dox food pellets in a microisolation rack system (FRP BIO2000, CLEA Japan) consisting of 16 individually ventilated boxes (1–4 cages/box) with glass fiber filters. Before starting behavioral experiment, the mice were kept under the condition of Dox withdrawal (OFF Dox) for 2 days and then exposed to context A for 6 min. One day later, the mice were subjected to contextual fear conditioning (CFC) in context B, consisting of 3 unsignaled foot shocks (2-s duration, 0.4 mA, 1 min apart) beginning 2 min after acclimation. After the last shock, the mice remained in the context for 1 min and were then returned to their home cages. One day later, the mice were anesthetized with approximately 2.0% isoflurane, and the dummy cannulas were replaced with two-branch-type optical fiber units comprising a plastic cannula body and a tightly connected 0.25-mm-diameter optic fiber (COME2-DF2-250; Lucir, Ibaraki, Japan). The tip of the optical fiber was targeted slightly above CA3 (DV, −1.5 mm from bregma). The mice were then returned to their home cages for 1–1.5 h. For the optical stimulation session, the mice were moved to the experimental room, and the fiber unit was connected to an optical swivel (COME2-UFC; Lucir), which was connected to a laser (200 mW, 473 nm, COME-LB473/200; Lucir) via a main optical fiber. The delivery of laser pulses was controlled by a schedule stimulator (COME2-SPG-2; Lucir) operating in time-lapse mode. The mice in their home cages were subjected to ten trains of laser pulses, each consisting of 300 500-μs pulses at 20-Hz of 473 nm light (approximately 10 mW output from the fiber tip) with 45-s intertrain intervals. Approximately 1–1.5 h after the laser stimulation, the mice were anesthetized with approximately 2.0% isoflurane, the optic fiber unit was detached, and the mice were again returned to their home cages. The mice were then given food containing 1,000 mg/kg Dox for 2 days and then maintained on food containing 40 mg/kg Dox.

To test fear memory, the mice were placed in contexts A and B for 3 min each at 1 and 2 days after the optical stimulating session, respectively. At the end of each session, the mice were returned to their home cages and the contexts were cleaned with water and 80% ethanol. A video tracking system (Muromachi Kikai, Tokyo, Japan) was used to measure the freezing behavior of the animals, as described in previous studies [10]. Freezing was defined as no movement detected for >1.5 s. All training and testing were conducted during the light phase of the light-dark cycle. The mean values of the freezing responses during each session were analyzed except for the context A pre-exposure session, for which freezing responses during first 3 min were analyzed. On day after context B test, mice were perfused and histologically analyzed.

**Histology**

Histological analysis was conducted as described previously [10, 11]. The mice were deeply anesthetized with an overdose of pentobarbital solution and perfused transcardially with 4% paraformaldehyde in phosphate-buffered saline (PBS; pH 7.4). The brains were removed and further post-fixed by immersion in 4% paraformaldehyde in PBS for 24 h at 4°C. Each brain was equilibrated in 25% sucrose in PBS and then frozen in dry-ice powder. Coronal sections of 30 μm were cut on a cryostat and transferred to 12-well cell culture plates (Corning, Corning, NY) containing PBS. After washing with PBS, the floating sections were treated with 4',6-diamidino-2-phenylindole (DAPI) (1 μg/ml, 10236276001; Roche Diagnostics) at room temperature for 20 min and then washed with PBS three times (3 min per wash). The sections were mounted on slide glass with ProLong Gold antifade reagent (Invitrogen of Thermo Fisher Scientific, Waltham, MA). Images were acquired on a fluorescence microscope (BZ9000; Keyence, Osaka, Japan) with a Plan-Apochromat 20× objective lens (Nikon, Tokyo, Japan). To quantify the number of ChR2-mCherry-positive cells, images of CA3 were acquired by collecting z-stacks (2.4 μm apart, 5–6 images). Maximum intensity projections of the images were created with the image analysis software (BZ-II; Keyence). Two sections (AP, approximately −1.9 and −2.0 mm from bregma) corresponding to each region of interest (ROI) (CA3 pyramidal cell layers from both hemispheres within 540 × 720 μm^2^) were chosen from each mouse, and the ChR2-mCherry-positive cells in the ROI were counted manually. The average number of ChR2-mCherry-positive cells per section from one hemisphere are presented throughout the text.

**Statistical analysis**

Statistical analyses were performed using GraphPad Prism 6 (GraphPad Software, Inc., La Jolla, CA). Comparisons of data between two groups were analyzed with two-sided Student’s *t* tests. Quantitative data are expressed as the means ± standard errors of the means (SEMs).
